# The Clinical Profile and Prognosis of Chinese Children with Melamine-Induced Kidney Disease: A Systematic Review and Meta-Analysis

**DOI:** 10.1155/2013/868202

**Published:** 2013-08-07

**Authors:** Pei-Xin Wang, Hong-Tian Li, Long Zhang, Jian-Meng Liu

**Affiliations:** ^1^Institute of Reproductive and Child Health/Ministry of Health Key Laboratory of Reproductive Health, School of Public Health, Peking University, 38 College Road, Haidian District, Beijing 100191, China; ^2^Department of Epidemiology and Biostatistics, School of Public Health, Peking University, 38 College Road, Haidian District, Beijing 100191, China; ^3^Guangxi Administration of Work Safety, 10 Dongbao Road, Qingxiu District, Nanning 530022, China

## Abstract

*Introduction*. Studies have reported inconsistent results regarding clinical feature and the prognosis status of the affected children in China melamine-contamination event. We summarized available literatures by performing a review and meta-analysis. *Methods*. Statistical pooling was performed using random-effects model; the sources of heterogeneity were explored through subgroup analyses. *Results*. Twenty-six studies involving 2164 patients with kidney abnormalities were identified; 94.4% of the patients had urinary calculi and 95.8% of the calculi were <10 mm in diameter. Of 2040 patients with known types of treatment, 5.6% underwent surgical treatment. The pooled recovery rates at 1, 3, 6, and 12 months after diagnosis or treatment initiation were 67.1%, 76.3%, 85.4%, and 92.3%, respectively; these pooled rates did not differ between the study subgroups stratified by mean age at diagnosis, mean duration of melamine exposure, types of patients (inpatient/outpatient), and treatment types (specific/nonspecific), except that the 1-month recovery rate for studies involving a specific treatment (71.9%) was higher than that for studies involving non-specific treatment (46.2%). *Conclusion*. The majority of patients had small calculi and could recover without surgical treatment. Kidney abnormalities remained in about 8% of the patients at 12-month followup, indicating a need for longer-term followup.

## 1. Introduction

In 2008, China experienced a melamine-contamination event, which has caused widespread concern and criticism [1–4]. Despite that the event was timely controlled, it was still estimated that approximately 300,000 children were diagnosed with kidney disease and over 50,000 patients received inpatient treatment [5]. During the past years, many studies have investigated the clinical characteristics and prognosis status of the affected children based on medical records from a single hospital or a few nearby hospitals. These studies have reported somewhat inconsistent results, probably because most of these studies had a small number of patients and because the disease severity of patients might also differ across studies.

Although the event has ended, it may still be valuable to provide an overview of the clinical profile and prognosis status of the affected children. There are three reasons. Firstly, the overall clinical and prognosis information may be useful for the treatment of melamine-induced or other similar renal diseases in the future. Young children are still likely to be exposed to melamine-contaminated dairy products, as supported by the fact that (1) the melamine-tainted milk powder reappeared in Chinese markets in 2010 [6] and (2) Irani investigator recently reported that the melamine concentration in totally nine different types of powder milk ranged from 1.5 to 30.3 *μ*g/g, which all exceeded the maximum limit of 1 *μ*g/g recommended by USFDA [7]. Secondly, the overall clinical information may provide clues to future experimental research into the mechanism and pathogenesis of melamine-related kidney damage. Thirdly, the overall prognosis information may be of particular importance for the Chinese authorities responsible for policy development and insurance management in relation to the affected children. Therefore, we conducted this systematic review and meta-analysis to summarize available literatures about the clinical profile and recovery status of the affected children.

## 2. Methods

### 2.1. Literature Search

PubMed and EMBASE were searched for relevant studies using the following three keywords: “melamine,” “cyanuramide,” and “1,3,5-triazine-2,4,6-triamine.” China National Knowledge Infrastructure and Wanfang database, the two major Chinese electronic databases, were searched for articles published in Chinese. These searches were limited to human studies published between September 2008 and June 2012. The reference lists of retrieved articles were hand-searched for additional studies. Moreover, we searched the PubMed monthly after June 2012 to identify any newly published studies.

### 2.2. Selection Criteria and Data Extraction

Studies were considered to be eligible for the review and meta-analysis if they reported a recovery rate for a specified period after diagnosis or initiation of treatment (i.e., 1 month, 3 months, and 6 months). A customized database was developed for data extraction. The information that was collected from each article included the following: the first author, the number of patients, types of patients, treatment types, follow-up duration, and recovery status; the mean and range of patients' age and duration of melamine exposure; the gender ratio of patients; types of kidney abnormalities; the clinical manifestation of patients; and the mean and range of diameter of calculi.

### 2.3. Quality Assessment

Because no standard instrument is available for quality assessment of follow-up studies [8], we developed a customized checklist with reference to previous meta-analyses [9, 10]. The checklist awarded each study a maximum of 10 scores from the following 5 aspects: sampling methods (population-based, hospital-based, and not reported was scored 2, 1, and 0, resp.), the description of melamine exposure and kidney abnormalities (clearly stated, partly stated, and not reported was scored 2, 1, and 0, resp.), the description of demographic characteristics (clearly stated, partly stated, and not reported), the description of therapeutic measures (clearly stated, partly stated, and not reported), and the rate of loss to followup (0%, <15%, and ≥15% was scored 2, 1, and 0, resp.) [11]. We defined ≥7 scores as high quality and <7 scores as low quality [12].

### 2.4. Statistical Methods

Data syntheses were performed using the Meta-Analyst 3.13 and RevMan 5.1. The pooled recovery rate, male to female ratio, percentage of asymptomatic patients, percentage of patients with calculi <10 mm in diameter, and their 95% confidence intervals were calculated based on random effects model, using the reciprocal of the variance as the weighting factor. Statistical heterogeneity across studies was assessed using the *I* ^2^ index; the value of 25%, 50%, and 75% was considered to be of low, medium, and high heterogeneities, respectively. Funnel plot and Begg's test were used for the assessment of publication bias. To assess the robustness of pooled recovery rates, we conducted sensitivity analyses by removal of low quality studies or excluding the study with the largest samples [13]. To explore the potential sources of heterogeneity, we performed various subgroup analyses according to mean diagnostic age of patients (≥18/<18 months), mean duration of melamine exposure (≥12/<12 months), types of patients (inpatient/outpatient), and treatment types (specific/nonspecific).

## 3. Results

### 3.1. Literature Search

**Figure 1:**
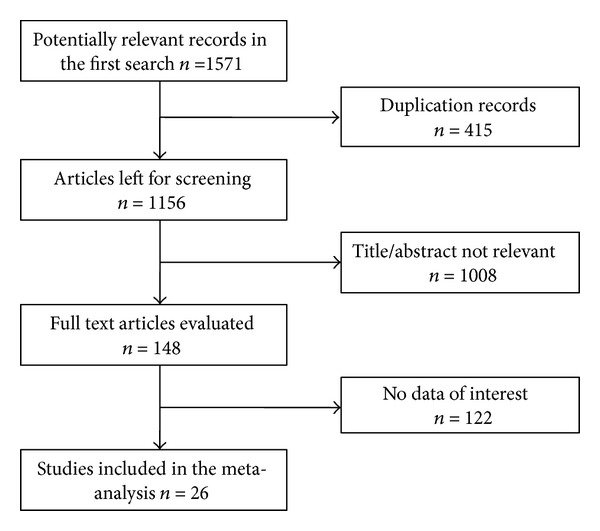
Process of study inclusion in meta-analysis.

Our initial search that was conducted on 30 June 2012 yielded a total of 1571 records (1188 in Chinese and 383 in English); after review of the titles/abstracts of 1156 nonduplicate records, we identified 148 potentially relevant records; after reviewing the full text, we identified 26 studies [14–39] that were eligible for the meta-analysis (Figure 1). Our subsequent search yielded one additional study [40] that was published after June 2012; although this study met the inclusion criteria, we did not included it in our meta-analysis because its results were rather contradictory to a previous study [14] that was conducted by the same study group.

### 3.2. Characteristics of Included Studies

The 26 studies, involving a total of 2164 patients, were conducted in 11 different provincial administrative areas of China, comprising 10 [14–17, 19–23, 38] published in English and 16 [18, 24–37, 39] in Chinese. The diagnosis and treatment of patients in all these 26 studies were according to the same standard recommended by the Chinese Ministry of Health [4]. The types of treatment primarily comprised surgical intervention, conservative treatment (i.e., taking Western drugs or traditional Chinese medicine, hemodialysis, and continuous renal replacement therapy), and only drinking more water; in this meta-analysis, we considered the former two types of treatment as receiving a specific treatment, whereas only drinking more water as receiving non-specific treatment. 

Of the 26 studies, 4 [23, 24, 28, 36] reported a separate recovery rate for different types of treatment or for different types of patients; in this meta-analysis, we treated each of these subgroup recovery rates as derived from different studies. Of these 26 studies, 12 [17, 18, 20, 21, 23, 24, 27, 30, 32, 33, 35, 38] were population-based and 14 [14–16, 19, 22, 25, 26, 28, 29, 31, 34, 36, 37, 39] were hospital-based; 3 [24, 27, 30] studies had a loss to follow-up rate ≥15%, 8 [14, 18, 22, 32–34, 37, 38] had a loss to follow-up rate ranging from 1% to 14%, and the remaining 15 [15–17, 19–21, 23, 25, 26, 28, 29, 31, 35, 36, 39] studies reported no loss to followup. In total, 22 were assessed to be of high quality [14–23, 25–32, 35–38] and 4 of low quality [24, 33, 34, 39]. Detailed characteristics and quality assessment results are shown in Table 1.

### 3.3. Clinical Characteristics

Of the 2164 patients, 2044 (94.5%) had urinary calculi, 103 (4.8%) had hydronephrosis, and 17 (0.7%) had urinary obstructions. Of the 26 studies, 13 [15, 17, 19, 24, 26–29, 31, 32, 34, 38, 39] reported the range of the calculi diameter, and the minimum diameter was 1 mm and the maximum 33 mm; 13 [15, 17–19, 23, 25, 26, 28, 32, 34–36, 38] reported the percentage of patients with calculi diameter ≤10 mm, and the pooled percentage was 95.5%. Of the 26 studies, 16 [14–17, 20–23, 25–28, 30–32, 38] clearly stated the clinical manifestation of patient, and the percent of asymptomatic patients ranged from 0 to 99.4%; the pooled percentage of asymptomatic patients was 76.2%. Of the 26 studies, 22 [14, 15, 17–22, 24–32, 34–38] reported the range of diagnostic age of patients, and the youngest age was 1.5 months and the oldest 120 months; 11 [17, 18, 20–22, 25, 26, 31, 32, 37, 38] reported the range of duration of melamine exposure, and the shortest duration of exposure to melamine was 0.5 month and the longest 69 months; 24 [14, 15, 17–38] reported the male to female ratio (ranging from 0.67 : 1 to 3.17 : 1), and the pooled ratio was 1.49 : 1. Of the 26 studies, 24 [14–19, 21–39] studies involving a total of 2040 patients clearly stated the types of treatment; 1502 received conservative treatment, 424 received non-specific treatment, and 114 received surgical treatment (including 44 patients whose surgery was performed after unsatisfactory conservative treatment).

### 3.4. Pooled Recovery Rates

Of the 26 studies, 15 reported a recovery rate at one month after diagnosis or initiation of treatment, 10 reported a recovery rate at 3 months, 7 at 6 months, 5 at 12 months, 1 at 18 months, and 1 at 24 months. The pooled recovery rates at 1, 3, 6, and 12 months were 67.1% (95% CI 57.1%–75.8%), 76.3% (95% CI 67.0%–83.6%), 85.4% (95% CI 78.6%–90.3%), and 92.3% (95% CI 83.6%–96.6%), respectively; the corresponding values of *I* ^2^ statistics were 47.2%, 46.4%, 42.7%, and 44.8% respectively, suggesting that a moderate level of heterogeneity existed across each set of individual studies. These pooled rates did not substantially change after excluding low quality studies (72.1%, 78.7%, 85.7%, and 94.9%, resp.) or excluding the study with the largest sample size (66.5%, 76.7%, 84.8%, and 90.9%, resp.). We further conducted subgroup analyses for the pooled recovery rates at 1, 3, and 6 months but not for that at 12 months due to the limited number of available studies. These pooled rates did not differ between subgroups stratified by mean age at diagnosis, mean duration of melamine exposure, types of patients, and types of treatment, except that the pooled rate in the first month for studies involving a specific treatment was significantly higher than that for studies involving non-specific treatment (71.9% versus 46.2%, *P* for interaction <0.001; Table 2). The study that reported a recovery rate at 18 months included a total of 91 patients and the recovery rate was 82.4% (95% CI 73.2%–88.9%); the studies that reported a recovery rate at 24 months included 195 patients and the recovery rate was 99.5% (95% CI 96.5%–99.9%).

### 3.5. Publication Bias

**Figure 2:**
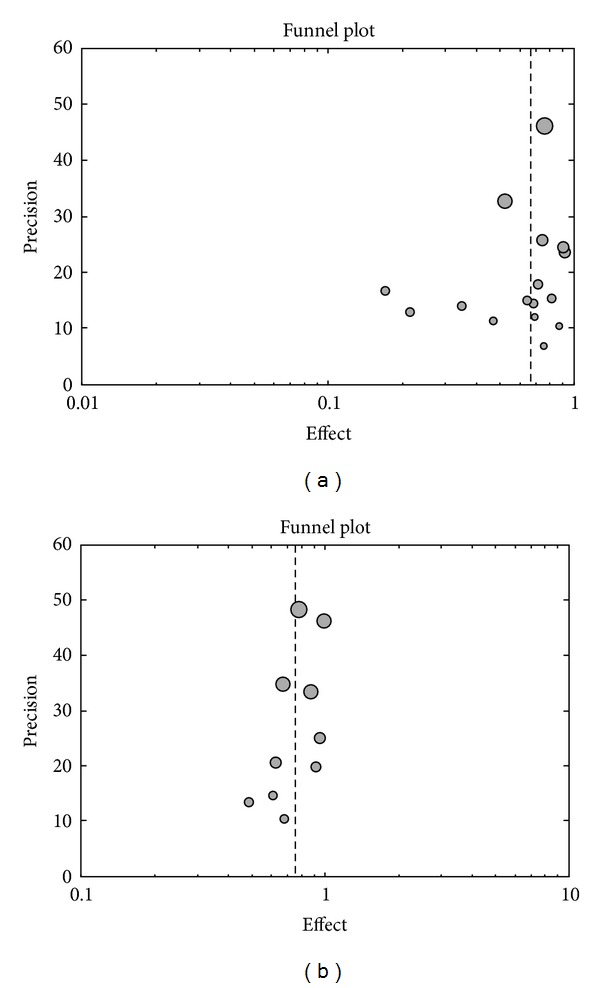
Funnel plots for the recovery rate at 1 month (a) and at 3 months (b).

The funnel plots regarding the recovery rates at 1 month and at 3 months were slightly asymmetric (Figure 2); however, corresponding Begg's test did not support the existence of publication bias (*Z *= 0.37, *P* = 0.711; *Z *= 0.89, *P* = 0.371, resp.). We did not generate a funnel plot and perform a formal statistical test for the recovery rates at 6 and 12 months due to the limited number of available studies [41].

## 4. Discussion

In this review and meta-analysis, we found that about 95% of the patients had urinary calculi and about 95% of the patients did not require surgical intervention. We also found that about 85% of the patients recovered in the first 6 months, whereas in the subsequent 6 months, only about half of the remaining 15% of patients recovered. We identified very few studies that reported a recovery rate for more than 12 months.

The summary of clinical characteristics showed that the majority of patients had urinary calculi, which corroborates the view that the melamine-related kidney abnormalities are primarily induced by physical obstruction of the calculi [42]. The summary of clinical characteristics also showed that 95.5% of the calculi had a diameter ≤10 mm, 76.2% of the patients were asymptomatic, and 94.4% of the patients did not require a surgical intervention, which further supports the general belief that melamine-resulted calculi are usually loose and easily expelled from the body. In the main analysis regarding the pooled recovery rate, we observed that about two-thirds of the patients could recover in the first month after diagnosis or treatment initiation; we noted a moderate level of heterogeneity existing across individual studies; in further subgroup analyses, we observed that the pooled recovery rate for studies involving a surgical or conservative treatment was significantly higher than that for studies involving non-specific treatment, suggesting that a timely treatment should be carried out in the early stage after diagnosis even when the patients were clinically asymptomatic. We also noted a low to moderate level of heterogeneity existing across studies that reported recovery rates at three months and at six months; however, in subgroup analyses, we observed that the heterogeneity for each of the two sets of studies could not be explained by mean age at diagnosis, mean duration of melamine exposure, types of patients, and treatment types, which remains to be studied further. Although the overall nonrecovery rate decreased over time, there was still 7.7% of the affected children who did not recover from kidney abnormalities at 12 months after diagnosis or treatment initiation; future studies need to explore potential genetic and/or environmental factors that affect the treatment effects. It should be noted that even a relatively lower non-recovery rate may be of practical significance in a public health perspective because the number of the affected children in the melamine contamination event was huge. Based on our pooled non-recovery rate, it is estimated that over 20,000 affected children did not recover from kidney abnormalities 12 months after diagnosis or treatment initiation. We are unable to provide an up-to-date overall estimate of the number of patients who did recover from the diseases because of the limited number of available studies. It may therefore be necessary to consider a longer-term follow up of the affected children.

To the best of our knowledge, this is the first meta-analysis to summarize the clinical profile and recovery status of the affected children. We synthesized 26 small-scale studies from one-third of the provincial administrative areas in China and provided some relatively robust estimates, such as pooled male to female ratio and pooled recovery rate. In addition to the main pooled analyses, we conducted various subgroup and sensitivity analyses to test the robustness of the pooled recovery rate and to explore potential sources of heterogeneity. Despite these strengths, our review has limitations. First, due to the lack of detailed information in the original reports, we were unable to conduct subgroup analyses by gender, diameter of calculus, consumption of milk powder, and disease severity; these subgroup findings may be of broad interest and clinical significance. Second, we selected studies primarily based on the availability of information on recovery status because we aimed to provide a complete clinical profile, especially the prognosis status, of the affected children. The strict selection criteria may have led to exclusion of studies that provided only the clinical characteristics of patients but with no information on recovery status.

## 5. Conclusions

This review and meta-analysis provides an overview of clinical profile and the recovery status of Chinese children affected by melamine-resulted urinary diseases. We found that the majority of patients had small urinary calculi, were asymptomatic or mildly symptomatic, and could recover from kidney abnormalities without surgical intervention. We also found that about 8% of the patients still had kidney abnormalities one year after diagnosis or treatment initiation. The lack of up-to-date data on recovery status of the affected children indicates the need for further investigation.

## Figures and Tables

**Table 1:** The characteristics of studies including in this meta-analysis.

First author	M/F ratio	Diagnostic age (month)*	Duration of exposure (month)*	Diameter of calculi (mm)	Percent of asymptomatic patients (%)	Patient types	Treatment types	Sample size	Follow-up duration (month)					Quality score
									1	3	6	12	24	
Sun [22]	2.13 : 1	11.8, 6–36	9.5, 0.5–24	—, —	0	IP	ST, CT	22	—	15	20	22	—	8
Chen [20]	1.88 : 1	24.0, 2–96	17.0, 2–69	—, —	87.8	OP	NO	49	—	30	—	—	—	8
Wen [15]	3.17 : 1	23.4, 2–84	12.4, —	<10, 100.0%	38.0	IP	CT	50	32	—	—	—	—	9
Wen [14]	2.61 : 1	25.5, 16–56	—, —	—, —	0	IP	ST, CT	195	—	—	—	—	194	7
Zhang [19]	0.67 : 1	16.0, 1–16	15.7, —	<5, 100.0%	—	OP	CT	54	49	—	—	—	—	7
Shen [16]	—	—, —	—, —	—, —	93.6	OP	DW	265	139	178	234	253	—	7
Yang [21]	3.00 : 1	18.0, 9–33	11.1, 5–18	—, —	0	IP	CT	8	6	—	—	—	—	10
Sun [23]	1.47 : 1	13.5, —	12.5, —	—, 94.9%	65.8	IP	CT	29	20	—	—	—	—	10
						OP	CT	50	46	—	—	—	—	
Kuang [32] and Gao [38]	1.82 : 1	19.0, 1–36	10.8, 1–19	1–72, 89.6%	73.3	IP	CT	96	—	—	66	—	75/91^$^	7
Liu [17]	1.61 : 1	19.8, 6–36	16.5, 2–36	1–9, 100.0%	91.5	OP	CT	47	—	—	42	—	—	8
Chang [33]	1.24 : 1	—, —	—, —	—, —	—	OP	DW	43	15	21	36	—	—	6
Zhang [28]	2.64 : 1	14.0, 2–36	—, —	5–15, 97.5%	68.8	IP	CT	37	30	—	—	—	—	8
						IP	ST	43	—	41	—	—	—	
Wang [30]	1.47 : 1	11.2, 2–66	—, —	—, —	99.4	OP	CT	389	295	306	—	—	—	7
Sun [25]	0.81 : 1	14.0, 2–96	11.0, 2–29	—, 76.9%	92.3	IP	CT	65	46	—	—	—	—	9
Tang [24]	1.68 : 1	—, 2–120	—, —	2–13, —	—	OP	CT	44	30	—	—	—	—	7
						OP	DW	30	14	—	—	—	—	
Zhang [39]	—	—, —	—, —	>4, —	—	OP	CT	41	7	—	—	—	—	6
Zhu [34]	2.15 : 1	15.6, 3–36	—, —	4–10, 100.0%	—	IP, OP	NO	75	—	—	—	59	—	5
Zhu [27]	0.77 : 1	9.2^#^, 2–66	—, —	3–20, —	98.2	OP	CT	28	6	—	—	—	—	7
He [26]	0.67 : 1	18.0, 5–36	—, 1–18	<5, 100.0%	73.3	OP	CT	60	54	—	—	—	—	9
Shang [36]	1.70 : 1	26.0, 13–48	—, —	—, 67.9%	—	IP	ST	27	—	—	—	25	—	8
						IP	CT	54	—	—	—	51	—	
Zhang [37]	1.78 : 1	16.0, 2–36	15.0, 2–36	—, —	—	IP	ST, CT	98	—	61/98	78/90	86/91	—	8
Mi [35]	2.00 : 1	44.4, 1–72	—, —	—, 95.0%	—	OP	CT	15	13	—	—	—	—	7
Long [31]	0.68 : 1	37.0, 19–72	14.0, 2–32	4–11, —	—	IP, OP	CT	37	—	34	—	—	—	8
Wang [29]	1.54 : 1	20.0, 3–36	—, —	4–19, —	80.3	IP, OP	CT	127	94	111	113	—	—	8
Xu [18]	1.55 : 1	20.4, 1–60	—, 1–36	—, 100.0%	—	OP	DW	86	—	86	—	—	—	9

M: male; F: female; IP: inpatient; OP: outpatient; ST: surgical treatment; CT: conservative treatment; DW: only drinking more water; NO: no description.

*The data was expressed as mean and range.

^#^The value was the median; in order to facilitate the calculation, we treated it as mean.

^$^The study [38] followed up the patients at 18 months after treatment initiation.

**Table 2:** Subgroup analyses regarding the pooled recovery rates (%) at 1, 3, and 6 months.

Subgroup	Rate (95% CI)		
	1 month	3 months	6 months
Treatment types			
Nonspecific treatment	46.2 (35.5–57.3)*	72.5 (48.7–88.1)	87.6 (83.4–90.8)
Specific treatment	71.9 (61.7–80.3)	81.5 (71.0–88.8)	85.2 (74.7–91.9)
Patient types			
Inpatient	70.5 (63.5–76.6)	77.6 (51.9–91.8)	82.1 (64.3–92.1)
Outpatient	64.5 (49.2–77.3)	71.2 (62.0–80.8)	87.8 (83.9–90.8)
Diagnostic age			
≥18 months	77.8 (66.1–86.3)	88.2 (68.1–96.3)	83.5 (65.5–93.1)
<18 months	74.7 (60.8–84.9)	76.5 (62.0–86.6)	87.4 (79.8–92.4)
Duration of exposure			
≥12 months	81.2 (62.8–91.8)	71.7 (52.8–85.2)	79.9 (48.2–94.4)
<12 months	71.2 (59.8–80.4)	68.2 (46.6–84.0)	87.5 (80.7–92.1)

*P value for test of heterogeneity between subgroup estimates was <0.001; all other *P* values for test of heterogeneity were >0.05.
